# Reduced Cortical Complexity in Children with Developmental Delay in Saudi Arabia

**DOI:** 10.7759/cureus.48291

**Published:** 2023-11-05

**Authors:** Abdullah H Abujamea, Mohammed Almosa, Mohammad Uzair, Nujud Alabdullatif, Shahid Bashir

**Affiliations:** 1 Department of Radiology and Medical Imaging, King Saud University Medical City, King Saud University, Riyadh, SAU; 2 Department of Radiology and Medical Imaging, King Saud University Medical City, King Saud University, Riyadh 12372, Saudi Arabia, Riyadh, SAU; 3 Department of Biological Sciences, Faculty of Basic & Applied Sciences, International Islamic University, Islamabad, PAK; 4 College of Medicine, King Saud University, Riyadh, SAU; 5 Department of Neuroscience, Neuroscience Center, King Fahad Specialist Hospital, Dammam, SAU

**Keywords:** magnetic resonance imaging, structural analysis, development delay, mri- magnetic resonance imaging, cortical thickness, brain structure

## Abstract

Introduction: Developmental delay (DD) is a neurodevelopmental disorder characterized by delays in multiple domains. The investigation of brain structure in DD has been enhanced by advanced neuroimaging techniques that can identify regional surface deformities. Neuroimaging studies have identified structural brain abnormalities in individuals with DD, but research specific to the Saudi Arabian population is limited. In this study, we examine the neuroanatomical abnormalities in the cortical and subcortical regions of Saudi Arabian children with DD.

Method: A T1-weighted, 1-mm-thick MRI was used to acquire structural brain images of 29 children with DD and age-matched healthy controls.

Results: Analysis of the MRI data revealed significant differences in several cortical and subcortical structures of gray matter (GM) and white matter (WM) in several brain regions of the DD group. Specifically, significant deformities were observed in the caudate nucleus, globus pallidus, frontal gyrus, pars opercularis, pars orbitalis, cingulate gyrus, and subcallosal gyrus. These findings suggest disrupted neurodevelopment in these regions, which may contribute to the cognitive, motor, and behavioral impairments commonly observed in individuals with DD.

Conclusions: The present study provides valuable insights into the neuroanatomical differences in Saudi Arabian children with DD. Our results provide evidence for cortical and subcortical abnormalities in DD. Deformities in the observed regions may contribute to cognitive impairment, emotional dysregulation, mood disorders, and language deficits commonly observed in DD. The structural analysis may enable the identification of neuroanatomical biomarkers to facilitate the early diagnosis or progression of DD. These results suggest that lower cortical complexity in DD children due to alterations in networks may play a critical role in early brain development.

## Introduction

Developmental delay (DD) is a complex disorder characterized by slower growth and progress in motor skills, language, cognition, and social interaction in children [1, 2]. Developmental delay significantly affects approximately 1%-3% of children under five years of age [3, 4], leading to substantial morbidity and mortality. However, the prevalence of DD might vary across different populations and geographic regions. In the Kingdom of Saudi Arabia (KSA), the prevalence of DD might range between 1.5% and 2.5% in children under two years of age due to a high rate of consanguinity [5, 6]. While DD can have various causes, including genetic factors [7], preterm birth [8], and environmental influences [9], understanding the structural brain abnormalities associated with DD is crucial for elucidating its underlying causes, progression, and effective interventions. Individuals with DD exhibit considerable morphological abnormalities in the brain, although the effect sizes and patterns of these differences vary across studies.

The human brain undergoes complex developmental processes during early life. Normative brain development is characterized by dynamic changes in brain regions, including cerebrum tissue, cortical regions, subcortical regions, ventricular volume, cerebrospinal fluid (CSF), gray matter (GM), and white matter (WM) [10]. These changes occur in an age-related manner and involve both short-range and long-range connections [11, 12]. This developmental remodeling of brain connectivity, often referred to as pruning and strengthening of network connectivity, contributes to hemispheric specialization, increased efficiency, and reduced redundancy in neural processes [13-15]. However, disruptions in these processes can contribute to the emergence of cognitive and behavioral impairments observed in individuals with DD [16-18]. Neuroimaging techniques, such as magnetic resonance imaging (MRI), functional MRI (fMRI), diffusion tensor imaging (DTI), etc., enable the investigation of the disrupted structural organization and functioning of the brain in DD [19-21]. Neuroimaging facilitates the study of regressive and progressive events underlying developmental delay etiology through the identification of neuroanatomical biomarkers [22]. Magnetic resonance imaging has emerged as a potential tool for investigating the structural organization and functioning of the brain [21]. An MRI provides non-invasive and high-resolution visualization of various brain regions and their connectivity, allowing researchers to study the anatomical and functional characteristics of the brain [23, 24]. Moreover, examination of the morphological characteristics of the brain in DD patients could provide an understanding of how developmental processes are disturbed.

The cortical areas, also known as cortical regions, are specific functional areas of the brain located within the cerebral cortex. The cerebral cortex is the outer layer of the brain and is composed of gray matter, responsible for higher cognitive functions and sensory processing [25]. The cortical regions are specialized for different higher-level functions, such as motor control, perception, memory, language, and emotional processing [26]. These regions work in concert to support various cognitive processes and are interlinked through neural networks. Moreover, the structural characteristics of cortical regions, with their distinct functional specializations, are closely linked to cortical thickness, which provides a measure of the underlying brain structure supporting cognitive processes [27]. Cortical thickness, referring to the thickness of the cerebral cortex, is commonly assessed using MRI [28, 29]. Cortical thickness is related to cognitive abilities such as attention, memory, and executive functions [30]. The study of cortical areas and cortical thickness is crucial for understanding brain development, cognition, and neurodevelopmental disorders. Examination of the regional variations in cortical thickness within specific cortical areas using neuroimaging modalities enables the understanding of the underlying mechanisms of normal brain development and the identification of potential markers of neurological disorders, including DD [28, 29, 31]. Magnetic resonance imaging techniques, such as T1-weighted imaging, can assess cortical thickness, while diffusion-weighted imaging can measure white matter integrity and connectivity. Therefore, this study on cortical region analysis compares multiple subcortical brain structure differences in the DD group and healthy controls in KSA. A high-resolution MRI technique was used to compare cortical complexity analysis in DD groups and healthy controls.

## Materials and methods

Participant acquisition

The study protocol was reviewed and approved by the ethical committee for scientific research at King Saud University, Riyadh, Saudi Arabia, based on the ethical guidelines of the 1975 Declaration of Helsinki under file number 22/0768/IRB. An informed consent was completed and signed by the child's parents or guardians before proceeding to the exam.

Demographic information, including age, gender, and relevant medical history, was collected for each participant. Throughout the study, strict adherence to ethical guidelines was maintained. The subject's personal information was anonymized to protect participant privacy.

Inclusion criteria

The healthy control groups consisted of typically developing children without any known developmental delays or neurological disorders. They were referred to an MRI for other reasons, and the brain scan was done following that procedure. They were within the age range of three to 10 years. The DD group included children diagnosed with DD, confirmed by a formal cognitive assessment conducted using the Griffith Mental Development Scale [5]. This comprehensive assessment covered various developmental areas, including locomotor skills, personal and social development, hearing and language abilities, eye and hand coordination, and performance and practical reasoning skills.

Exclusion criteria

Individuals with a diagnosis of any known genetic, neurological, or medical conditions affecting neurodevelopment, such as autism, cerebral palsy, or genetic syndromes, were excluded. Patients with a history of significant head trauma or brain injury were also not included. Additionally, individuals with contradictions for MRI scanning (e.g., presence of metal implants, claustrophobia), too sick to undergo MRI, or non-cooperative individuals for the developmental assessment were not included to ensure reliable data collection. Participants with incomplete data sets were also excluded from the analysis to ensure data integrity and consistency.

Anatomical image acquisition and processing

Magnetic Resonance Imaging Structural Image Acquisition

Magnetic resonance imaging structural images were acquired using a Siemens Magnetom Verio 3T MRI clinical scanner (Siemens AG, Healthcare Sector, Erlangen, Germany) equipped with a 12-channel phased-array head coil. The T1-weighted 3D magnetization-prepared rapid gradient-echo imaging (MPRAGE) was employed for image acquisition. The imaging parameters used were as follows: repetition time (TR) = 1600 ms, echo time (TE) = 2.19 ms, inversion time = 900 ms, flip angle = 9°, acquisition plane = sagittal, voxel size = 1 × 1 × 1 mm3, field of view (FOV) = 256 mm, acquired matrix = 256 × 256, and an acceleration factor (iPAT) of 2.

Data Analyses

All MR imaging processing and analysis were performed using the BrainSuite software (Version 15b; http://brainsuite.org/). BrainSuite offers a range of automated image analysis tools specifically designed for neuroimaging. The processing pipeline in BrainSuite involved several steps to extract surface models of the cerebral cortex from the 3D T1 MRI images. These steps included skull and scalp removal, non-uniformity correction, tissue classification, registration-based identification of the cerebrum, topology correction, and surface generation. These procedures resulted in the creation of triangular surface mesh models representing the inner and outer boundaries of the cerebral cortex.

To ensure accurate coregistration between the subject’s brain and an atlas, the BrainSuite software utilized both surface models and volumes of the brain images. This approach incorporated anatomical information from the surface models and volume data to achieve alignment [32]. Furthermore, BrainSuite employed a final parcellation that computed volumes for 140 brain regions, with 70 regions in each hemisphere. This parcellation allowed for the quantification of brain regions of interest. For each brain area, surface and volume registration (SVReg) was utilized to generate volumes for the WM, GM, and CSF.

Statistical Analysis

A statistical analysis was performed to compare the cortical thickness measurements between healthy controls and DD groups. All analyses were performed on IBM SPSS statistics, version 22 (IBM Corp., Armonk, NY). Group differences were assessed using an appropriate statistical test, i.e., a t-test. P <0.05 was considered statistically significant.

## Results

Study participants

Demographic characteristics of the study participants are presented in Table 1.

**Table 1:** Demographic data of participants included in the study DD: developmental delay

Parameter		Control (29)	DD (29)
Age (years)	Mean ± SD	6.1±2.8	4.7±1.6
	Median	5	4
	Range	3-10	3-9
Gender, n (%)	Female	15 (51.7%)	21 (72.4%)
	Male	14 (48.3%)	8 (27.6%)
BMI (kg/m^2^)	Mean ± SD	17.9±3.3	15.9±2.9

The DD groups and healthy control groups consisted of 29 individuals in each group. The mean age of the control group was 6.1 years (three to 10 years), whereas the DD group had a slightly younger mean age of 4.7 years (three to nine years). Regarding gender distribution, the control group consisted of 15 females (51.7%), while the DD group had a higher proportion of females, i.e., 21 (72.4%). Body mass index (BMI) data were also collected for the participants. The control group had a mean BMI of 17.9 kg/m^2^, while the DD group had a slightly lower mean BMI of 15.9 kg/m^2^.

Cortical thickness analyses

**Figure 1:**
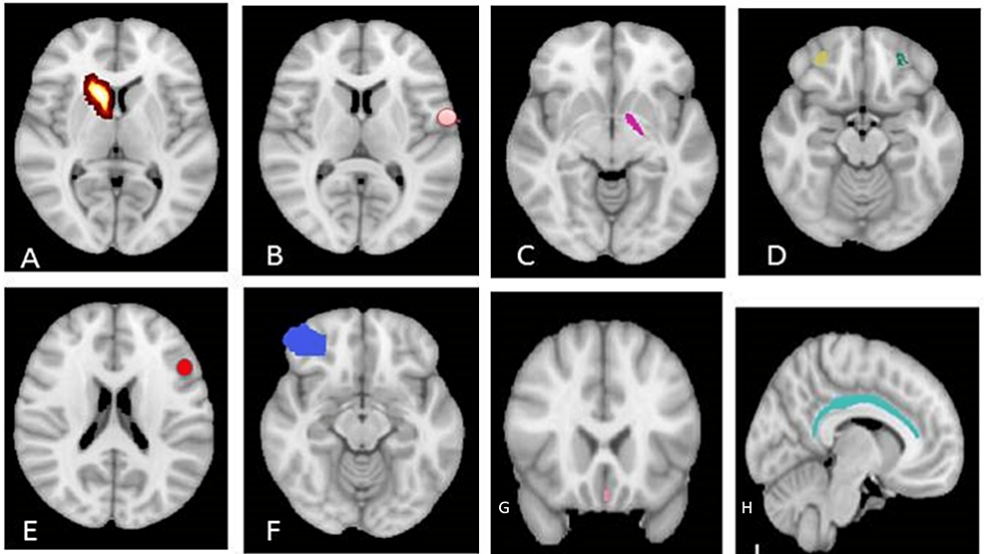
Developmental delay showed significant deformities in the following cortical regions: (A) right caudate nucleus, (B) left pre-central gyrus (WM), (C) globus pallidus, (D) left and right middle frontal gyrus (GM), (E) left pars orbitalis (GM), left pars opercularis (GM), (F) right middle frontal gyrus (WM), (G) left subcallosal gyrus (GM), and (H) left cingulate gyrus (GM).

The results of the cortical thickness analysis revealed significant differences in specific cortical regions between the DD and the control group. In the DD group, there were significant deformities observed in various cortical regions compared to the control group (Figure 1).

These regions include the right caudate nucleus (p<0.043), left globus pallidus (p<0.046), right middle frontal gyrus (GM) (p<0.012), left middle frontal gyrus (GM) (p<0.018), left pars opercularis (GM) (p<0.037), left pars orbitalis (GM) (p<0.042), left cingulate gyrus (GM) (p<0.041), left subcallosal gyrus (GM) (p<0.002), right middle frontal gyrus (WM) (p<0.031), left pars orbitalis (WM) (p<0.001), and left pre-central gyrus (WM) (p<0.019) (Figure 1, Table 2).

**Table 2:** Cortical thickness analysis of the control group and developmental delay group

Parameter	Control, mm (Mean ± SD)	DD, mm (Mean ± SD)	P-value
Right caudate nucleus	4.16±.776	3.80±.540	.043
Left globus pallidus	1.13±.143	1.11±.204	.046
Right middle frontal gyrus	16.79±4.31	16.06±2.88	.012
Left middle frontal gyrus	18.13±4.27	17.84±2.51	.018
Left pars opercularis	5.31±1.08	5.19±.667	.037
Left pars orbitalis	2.36±.907	2.33±.656	.042
Left cingulate gyrus	14.25±2.57	13.34±1.65	.041
Left subcallosal gyrus	.84±2.12	.18±0.71	.002
Right middle frontal gyrus	6.63±2.06	5.77±1.32	.031
Left pars orbitalis	.64±.237	.49±.096	.001
Left pre-central gyrus	7.02±1.61	6.37±1.04	.019

These findings suggest that individuals with DD exhibit significant alterations in the cortical thickness of specific brain regions compared to typically developing individuals. The affected regions involve both gray and white matter structures, highlighting the complexity of the observed abnormalities. The significance of these structural differences may be indicative of underlying neurodevelopmental disturbances associated with DD.

## Discussion

We found significant differences in the subcortical structures for GM and WM for the caudate nucleus, globus pallidus, frontal gyrus, pars opercularis, pars orbitalis, and cingulate gyrus (GM) in Saudi DD patients. The observed deformities in the cortical regions of individuals with DD have significant implications for their neurodevelopmental outcomes. In a study on Iranian [33] and Indian [12] children with developmental delay, a significant proportion (20%) of the children were found to have brain structural abnormalities. The cortical region deformities may contribute to the cognitive, motor, and behavioral impairments commonly observed in individuals with DD.

Research has shown that the caudate nucleus and globus pallidus play critical roles in motor control, coordination, cognition, and decision-making [34, 35]. Abnormalities in these regions can lead to motor deficits, including fine motor skills, coordination, cognitive impairment, and movement planning. The middle frontal gyrus is involved in various cognitive processes, such as attention, working memory, and executive functions [36]. The deformity of the middle frontal gyrus may result in cognitive impairments, including difficulties with attention, problem-solving, and decision-making. The cingulate gyrus is associated with emotional processing, regulation, and social cognition [37]. The abnormalities in the cingulate gyrus were also reported in patients with attention-deficit hyperactivity disorder (ADHD) [38, 39]. Similarly, the subcallosal gyrus is also associated with mood regulation and emotional processing [40]. Structural alterations in these regions may contribute to emotional and social difficulties, such as challenges with emotional dysregulation, social isolation, mood disorders, sadness, fear, and empathy. The precentral gyrus, located in the frontal lobe, is responsible for motor control and the initiation of voluntary movements [41]. A deformed precentral gyrus could cause motor impairments, including difficulties with coordination, motor planning, and execution. The pars orbitalis and pars opercularis are regions within the frontal operculum, which is involved in language processing and production [42-44]. Abnormalities of the pars orbitalis and pars opercularis may lead to language and communication difficulties, such as speech initiation disturbances, expressive language deficits, impaired language processing, and challenges with articulation and fluency.

Gray matter represents the neuronal cell bodies, dendrites, and synapses, and it plays a vital role in information processing and cognition. Deformities in observed GM regions, including the middle frontal gyrus, pars opercularis, pars orbitalis, cingulate gyrus, and subcallosal gyrus, could disrupt attention, working memory, language processing, and emotional regulation. White matter, on the other hand, consists of myelinated axonal fibers that facilitate communication between different brain regions [45]. Disruptions in the WM regions, including the middle frontal gyrus, pars orbitalis, and precentral gyrus, can affect the integrity and efficiency of neural connections, leading to impaired information transfer and coordination among brain regions [46]. These disrupted processes also contribute to motor deficits, language impairments, altered cognitive processing, and impaired visual memory [47, 48].

The findings of the present study, conducted in Saudi Arabia, are in line with previous research conducted in various countries, demonstrating consistent patterns of cortical abnormalities in individuals with DD [12, 34]. Abnormal findings on the MRI brain of patients with DD have been reported in numerous studies [5, 12, 49, 50, 51]. These abnormalities may include cortical abnormalities, CSF alterations, dysplasia, abnormal size or signal in the basal ganglia, and anomalies in the cerebellum and brain stem [52, 53]. The anatomically altered ventricles and WM, mainly the corpus callosum, are also reported to be associated with DD [5, 51, 54]. The present study also reported abnormalities in WM regions. Sun et al. (2022) reported that children with DD exhibited increased cortical thickness with lower surface area in asymmetric cortical regions, including pars orbitalis, pars operculais, and posterior cingulate [55]. Therefore, our study contributes to the growing body of literature on cortical abnormalities in individuals with DD, and the findings are consistent with previous studies conducted in different countries. The similarities across different populations suggest the presence of shared neurobiological mechanisms underlying DD, regardless of cultural or geographic differences.

The deformities in the cortical regions have significant implications for neurodevelopmental outcomes, including cognitive impairment, emotional dysregulation, mood disorders, and language deficits commonly observed in DD. These findings contribute to our understanding of the neurobiological underpinnings of DD and provide insights into the potential mechanisms that give rise to the observed impairments in individuals with DD.

Limitations and future perspectives

The present study has several limitations that should be considered when interpreting the results. First, the comparison between children with DD and healthy subjects makes it difficult to directly conclude the effects of cortical thickness on DD. A more ideal approach would involve a longitudinal study design to better understand the relationship between cortical thickness and clinical outcomes. Second, the subject group in our study was not homogeneous in terms of DD type, which may have introduced variability and obscured the interpretation of results. Future studies could benefit from a more homogenous sample to provide clearer insights into specific DD subtypes. Third, the small sample size of our study limits the statistical power and generalizability of the findings. Conducting larger-scale studies with a diverse population would enhance the robustness of the results. Lastly, it is important to note that caution should be exercised when extrapolating the findings of healthy subjects to patients, as patients with DD may have unique characteristics and heightened susceptibility for improvement, as demonstrated in our study. Further research is warranted to address these limitations and explore the underlying mechanisms of structural brain abnormalities in DD, ultimately leading to improved diagnosis, intervention, and outcomes for affected individuals.

## Conclusions

To the best of our knowledge, this is the first study conducted in the Kingdom of Saudi Arabia to investigate structural brain abnormalities in children with developmental delays using MRI imaging. This study revealed significant deformities in subcortical structures in cortical regions, as well as differences in GM and WM in the brain MRIs of children with DD. The observed deformities in the cortical regions of individuals with DD suggest disruptions in the structural organization and connectivity of the brain, highlighting the neurobiological underpinnings of DD. These disruptions may affect the efficient transmission of neural signals, leading to functional deficits in various critical cognitive, motor, and socio-emotional domains. The significant deformities in subcortical structures and cortical regions identified in our study highlight the importance of these regions in motor control, coordination, cognition, attention, language processing, emotional regulation, and social cognition. Furthermore, the consequences of these deformities may vary among individuals with DD, as the specific regions affected and the extent of the deformities may differ. The severity and nature of the DD can influence the manifestations and impact of these deformities. Moreover, understanding the specific cortical regions and their associated functions that are affected in individuals with DD can inform targeted interventions and therapies to address the unique challenges faced by these individuals. Early identification of these structural abnormalities through neuroimaging techniques may facilitate early interventions and support for individuals with DD, leading to improved outcomes and quality of life.
